# Small flexible structure for targeted delivery of therapeutic and imaging moieties in precision medicine

**DOI:** 10.18632/oncotarget.8335

**Published:** 2016-03-24

**Authors:** Shaofan Hu, Wei Wang, Yanling Zhang, Bingjie Li, Xiuchun Qiu, Chaoxia Zou, Henry Ran, Fujun Zhang, Shi Ke

**Affiliations:** ^1^ UTHealth, School of Public Health, Houston, Texas, USA; ^2^ Jiangxi Children's Hospital, Nanchang, China; ^3^ Baylor College of Medicine, Houston, Texas, USA; ^4^ School of Biotechnology, Southern Medical University, Guangzhou, China; ^5^ Sun Yat-sen University Cancer Center, State Key Laboratory of Oncology in South China, Collaborative Innovation Center for Cancer Medicine, Guangzhou, China; ^6^ The Fourth Military Medical University, Xi'an, China; ^7^ Harbin Medical University, Harbin, China

**Keywords:** precision medicine, target-specific imaging, target-specific therapy, molecule imaging, molecule therapy

## Abstract

The goals of precision medicine are to link diagnostic and therapeutic agents, improve clinical outcomes, and minimize side effects. We present a simple, small, flexible three-armed core structure that can be conjugated to targeting, imaging, and therapeutic moieties. The targeting molecule can be a peptide, protein, or chemical compound. The diagnostic reporter can be optical and/or nuclear in nature, and can be replaced by chemo- and/or radiotherapeutic compounds for treatment using a single targeting molecule. Imaging components can be used to detect disease biomarkers, monitor treatment response, and guide surgery in real-time to create a tumor-free margin. Isotope impurity can be exploited to visualize whole-body distribution of therapeutic agents. The one-to-one ratio of targeting component to therapeutic agents facilitates dose calculation. The simple synthesis and flexible, modular nature of the agent facilitate high-purity, large-scale production. The core capacity to “seek, treat, and see” may advance precision medicine in the future.

## INTRODUCTION

The goals of precision medicine are to improve clinical outcomes and minimize side effects. Advances in precision medicine identify and link diagnostic and treatment options [1]. Molecular imaging can provide information on disease status and treatment response at the cellular and molecular levels. Use of different reporters permits visualization of molecular changes from the level of the whole body (nuclear reporters) to single cells (optical reporters). Highly sensitive and specific targeting components can guide choice of chemo-, radio-, or surgical therapies. Therefore, by replacing diagnostic reporters with therapeutic agents, molecular imaging agents can be readily converted to target-specific drugs that treat disease locally at the cellular and molecular level, minimizing systemic toxicity. This switch links diagnostic and treatments options, improving both.

Here, we introduce a simple three-armed core structure to fulfill precision medicine needs. One arm is conjugated to a targeting molecule, which can be an antibody, peptide, cytokine, or small molecule, and directs the complex to a specific disease marker. The other two arms can be used for imaging, therapy, or a combination. For imaging purposes, one arm can be labeled with nuclear reporters (positron emission tomography [PET] or single-photon emission computed tomography [SPECT] isotope) and the other with optical imaging reporters, such as near infrared dye. Nuclear imaging is the gold standard modality for whole-body scanning. Optical imaging provides high spatial resolution for real-time, imaging-guided surgery aimed at achieving a tumor-free margin. For therapeutic purposes, one arm can be conjugated to cytotoxic β ray-emitting therapeutic isotopes and the other with cytotoxic chemotherapeutic agents, constituting a target-specific chemoradiotherapeutic agent. For combined imaging and therapeutic purposes, several combinations can be generated, depending upon the disease and the patient population.

For pediatric patients, one arm can be conjugated to a chemotherapeutic agent and the other to a long-wavelength optical reporter. This approach would eliminate radiation exposure in these radiosensitive patients and prevent occurrence of radiation-induced secondary cancer. These optical reporters have long penetration depth and are readily detected due to the patients' relatively small bodies.

For adult patients with chemosensitive disease, one arm can be labeled with a chemotherapeutic agent and the other with nuclear imaging isotopes. PET/SPECT nuclear imaging can be used to define dose, administration schedule, biodistribution, and treatment response. This imaging modality will serve as a gold standard for imaging validation by quantitative autoradiography followed by histology.

For adult patients with radiosensitive disease, two-armed structures can be used. The structure can be labeled with a single targeting component and the preparation divided into two pools. One pool can be labeled with a therapeutic isotope, and the other with an imaging isotope. Because the structures of the agents in the two pools are identical, with the exception of the reporter, the imaging results will be representative of the binding and distribution of the therapeutic agent and the tumor response.

For patients requiring chemotherapy for local control and surgical resection, the strategy used for pediatric patients will be applied, but with a shorter-wavelength optical reporter to improve spatial resolution. This complex could then be used in combination with a surgical microscope to remove the tumor mass, leaving a tumor-free margin [2–3].

## RESULTS

### Design of combined targeting/imaging/therapeutic agents for precision medicine

**Figure 1:**
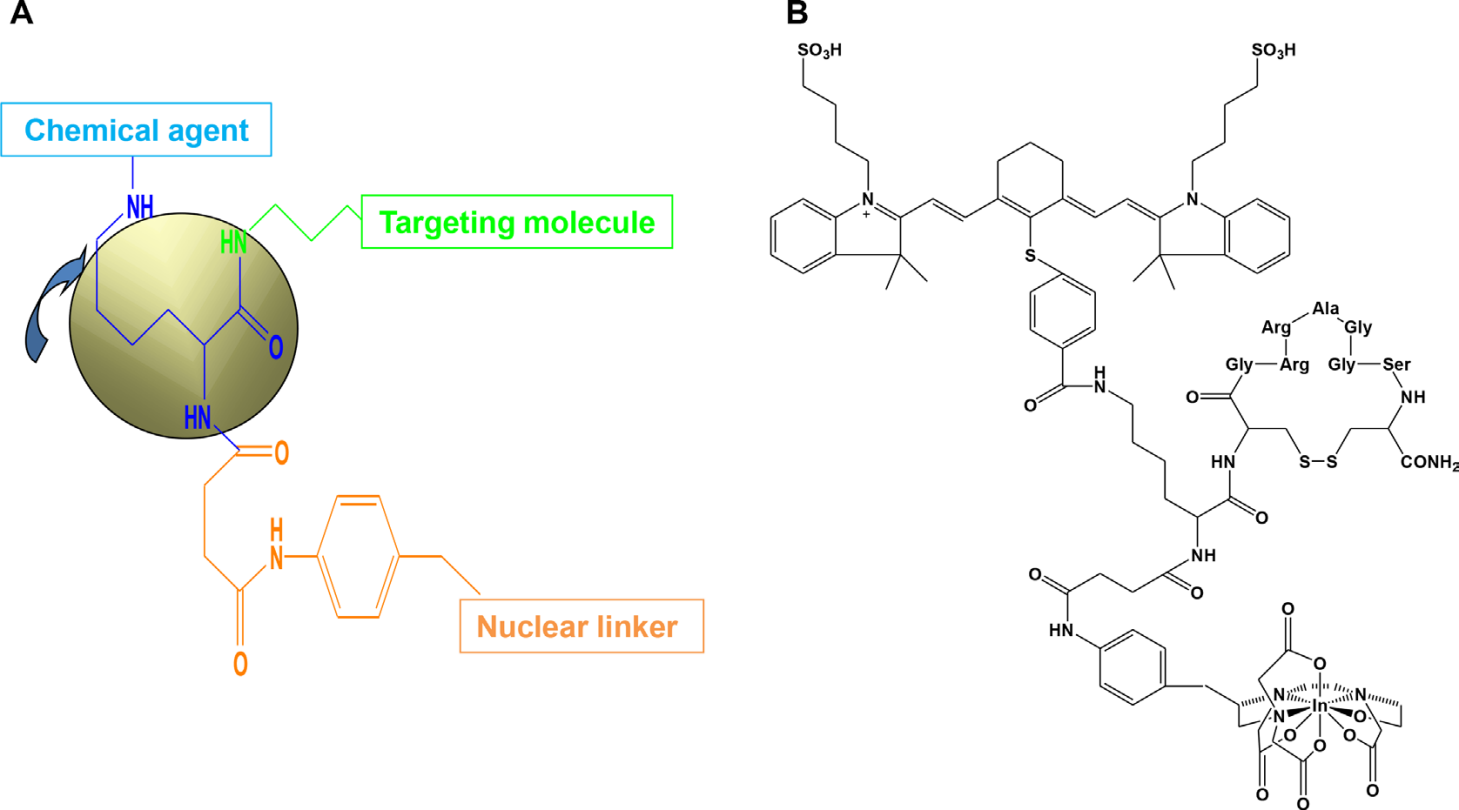
Core structure and final imaging agent The core structure contains three arms (**A**) for attachment of chemical agent, targeting molecule, and nuclear linker. Target molecule can be a peptide, protein, cytokine, chemokine, antibody, or chemical compound. The length of the targeting arm is very important for stable binding; if it is too long, stability of the agent will be reduced. The sequence and length should be customized and tested for each marker. The chemical arm can carry an optical reporter for diagnostic or chemotherapeutic purposes. The wavelength of the optical reporter can be changed as needed. Long-wavelength reporters have high penetration depth, and short-wavelength reporters have high spatial resolution. A molecule capable of being endocytosed can transport the agent intracellularly, and a cell-surface-binding molecule can function outside the cell. The nuclear linker arm can be conjugated to different nuclear linkers, depending on the nature of the isotope appropriate for the final purpose. An example of a core structure conjugated to a peptide optical interleukin (IL)-11-receptor-targeting molecule and SPECT nuclear imaging component is shown in (**B**). The optical reporter is a near-infrared dye with excitation and emission wavelengths of 785 and 830 nm, respectively. The nuclear chelator is diethylenetriaminepentaacetic acid (DTPA), and the isotope is indium-111.

Figure 1 shows the three-armed core structure (Figure 1A) and one example of an agent designed for imaging purposes (Figure 1B). Synthesis and characterization of this agent were described previously [4].

### Multiple target molecules for diagnostic molecular imaging

**Figure 2:**
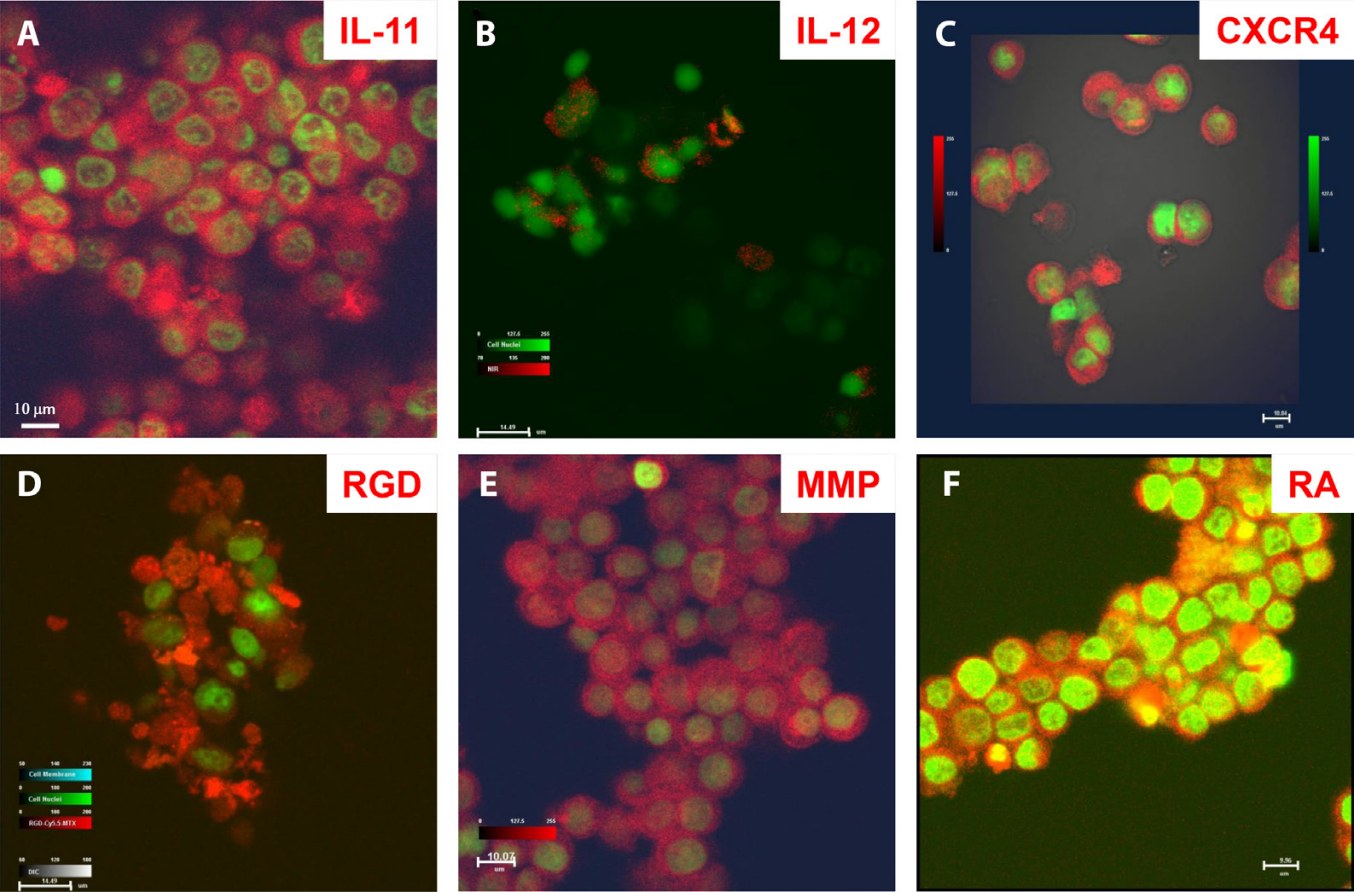
Disease marker detection at the cellular level by core structure carrying optical reporters Confocal microscopic images of target-specific agents bound to different compartments of cultured human F4 osteosarcoma cells. (**A**) Peptide agent targeting interleukin (IL) -11 was located at the cell surface and in cytosol and nucleoplasm, but not the nucleolus. (**B**) Peptide agent targeting IL-12 was bound to the cell surface only. (**C**) Peptide agent targeting cytokine CXCR4 was located at the cell surface and in cytosol. (**D**) Peptide agent targeting recognition sequence Arg-Gly-Asp (RGD) was located at the cell membrane and in cytosol, with uneven distribution depending on direction of cell migration. (**E**) Peptide agent targeting matrix metalloproteinase (MMP) was located in the cytosol. (**F**) Agent targeting retinoic acid (RA) was located at the cell membrane, in cytosol, nucleoplasm, and nucleolus (yellow).

**Figure 3:**
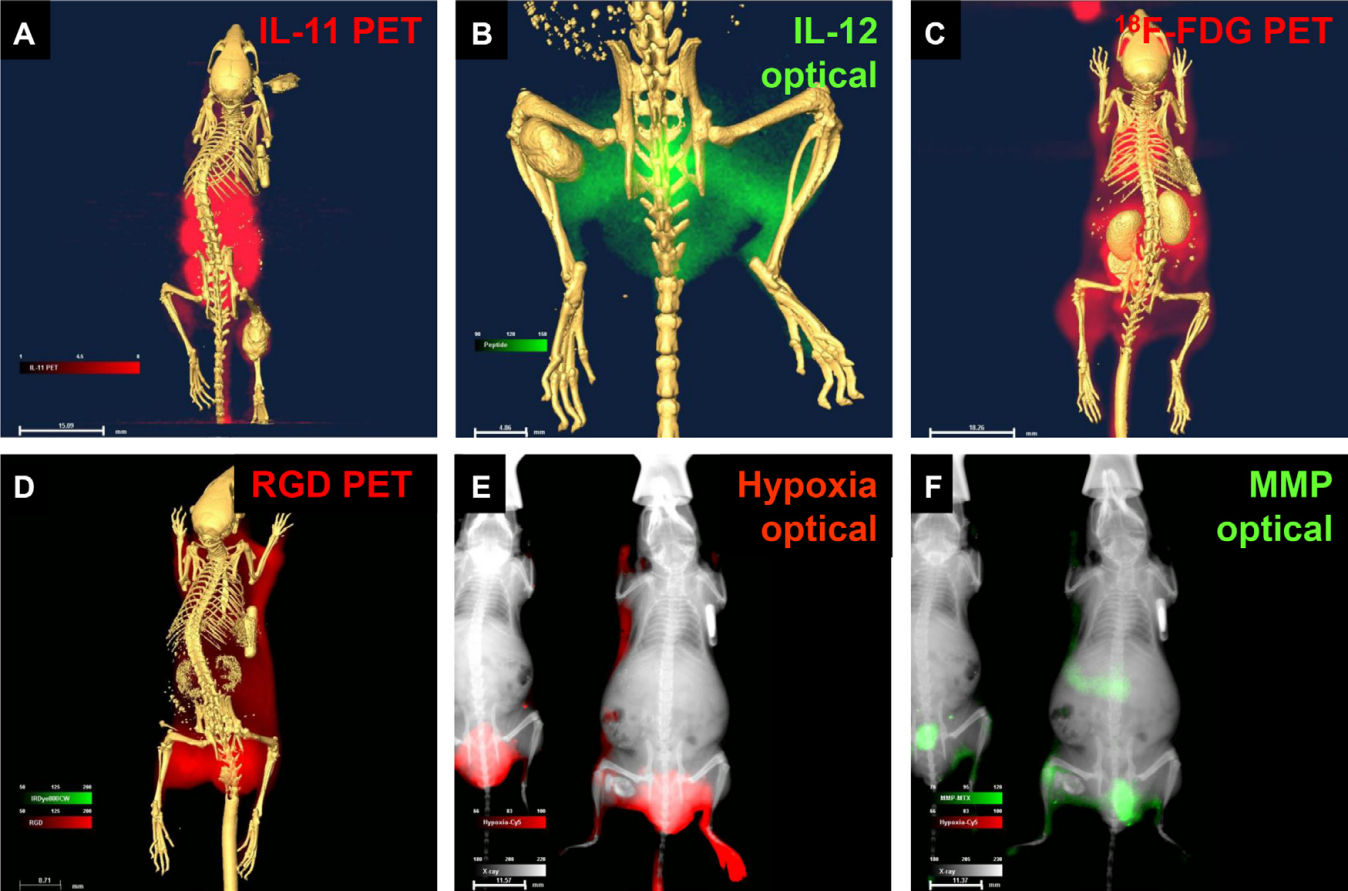
Core structure carrying reporters detected disease markers in living animals bearing osteosarcoma tumors (**A**) Peptide agent targeting IL-11 was detected by positive emission tomography (PET)/computed tomography (CT). (**B**) Peptide agent targeting IL-12 was detected by optical/CT. (**C**) ^18^F-FDG glucose was detected by PET/CT. (**D**) Agent targeting recognition sequence Arg-Gly-Asp (RGD) was detected by PET/CT. (**E**) Chemical indicator of hypoxia was detected by optical/X-ray (E). Peptide agent targeting matrix metalloproteinase (MMP) was detected by optical/X-ray (**F**).

To demonstrate the diagnostic capability of these agents at the cellular to whole-body level, we tested multiple agents in human osteosarcoma cells *in vitro* and *in vivo* and in a mouse model. Figure 2 shows peptide (Figure 2A–2E) and chemical (Figure 2F) targeting agents binding to cultured osteosarcoma cells. Whole-body biodistribution of these agents, based on detection using combinations of PET, computed tomography (CT), X-ray, and optical methods is shown in Figure 3A–3F.

### Imaging-guided surgery

**Figure 4:**
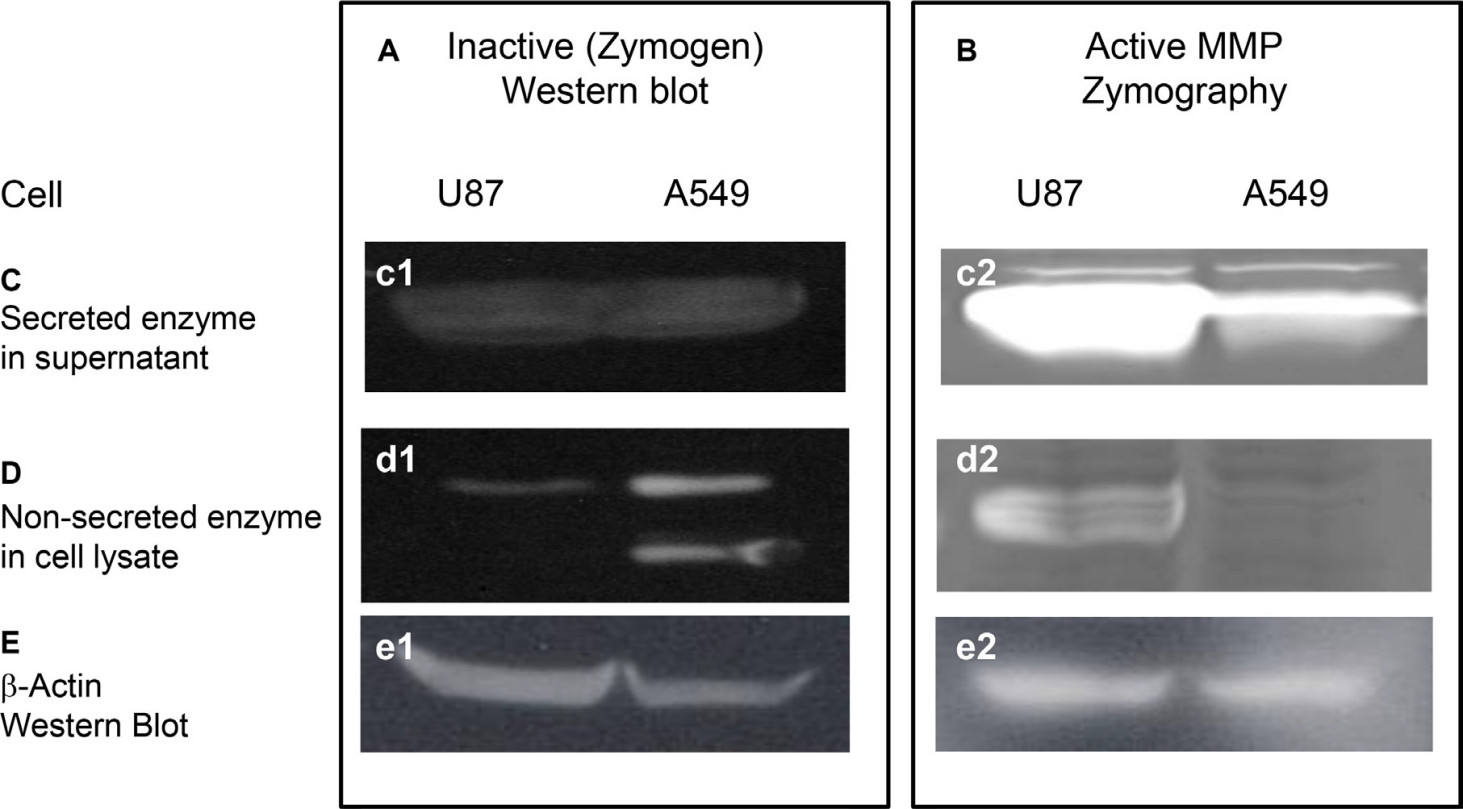
Western and zymographic analysis of zymogen and active matrix metalloproteinase (MMP) in human cancer cell lines Western (**A**) and zymographic (**B**) analysis of zymogen and active matrix metalloproteinase (MMP), respectively, in the supernatants (panels **C**) and lysates (panels **D**) of cultured U87 human glioblastoma/astrocytoma and A549 human lung carcinoma cell lines. Both cell lines showed similar levels of secreted zymogen in the supernatant (c1). U87 cells had a higher level of active MMP than A549 cells (c2). U87 cells had less intracellular zymogen than A549 cells in cell lysates (d1), but the level of active MMP in U87 cells was greater than that in A549 (d2). Western analysis of β-actin (**E**) confirmed that equal amounts of protein were loaded in all lanes.

To demonstrate the feasibility of using these agents in imaging-guided surgery, we conjugated a peptide targeting the active form of matrix metalloproteinase (MMP) and a near infrared optical reporter (IRDye800CW) to the core structure for application in mouse models of human glioma and lung carcinoma. We chose MMP as a target because it is secreted and required by cancer cells for progression/metastasis [5]. However, we first determined the levels of secreted and intracellular, inactive (zymogen) and active forms of MMP in U87 human glioma and A549 human lung carcinoma cell cultures by western blot analysis and zymography (Figure 4A–4E). The data show that U87 cells both secrete and retain intracellularly more active MMP and less zymogen than A549 cells.

**Figure 5:**
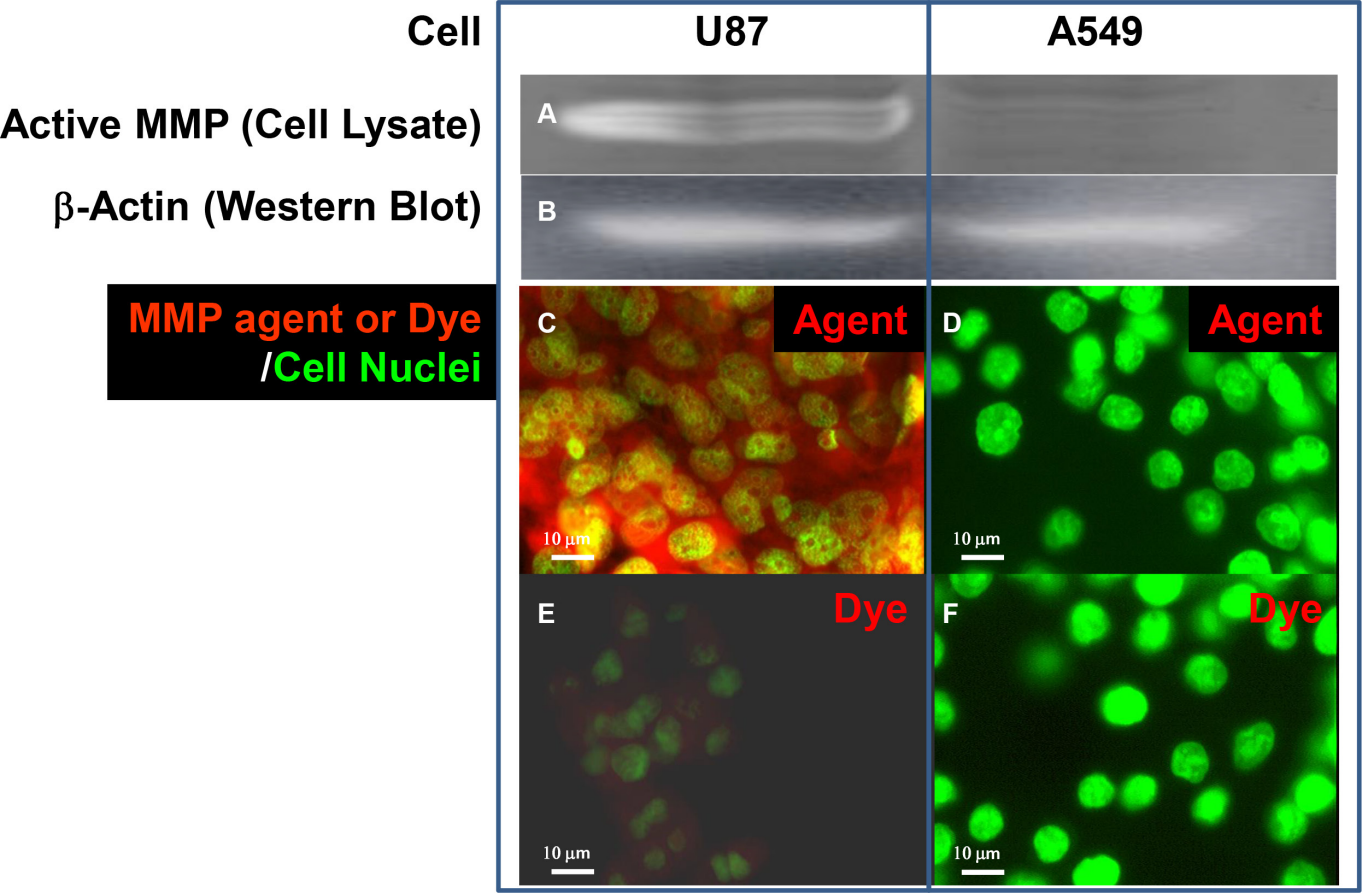
Detection of active MMP by zymography and with MMP-targeting agent Detection of active MMP by zymography in culture fractions (**A**) and in cultured U87 and A549 cells (**C** and **D**) by MMP-targeting agent. Zymography (a) showed that the U87 cell lysate contained more active MMP than that of A549 cells when (**B**) β-Actin analysis confirmed that an equal amount of protein was loaded in both lanes. The agent consisting of MMP-targeting moiety and IRDye800CW optical reporter conjugated to core structure bound specifically to U87 (C) but not A549 (D) cells. Free dye was detectable and showed a low level of nonspecific staining in active-MMP-positive U87 cells with overexposure (**E**). Free dye was undetectable in active–MMP-negative A549 cells under the same exposure conditions (**F**). Nuclei were stained with Sytox Green.

We confirmed the zymographic findings (Figure 5A and 5B) and demonstrated much stronger binding of the MMP-targeting agent to cultured U87 (Figure 5C and 5E) than to A549 (Figure 5D and 5F) cells. These findings demonstrate that our MMP-targeting agent specifically targets the cells that show high levels of active MMP.

**Figure 6:**
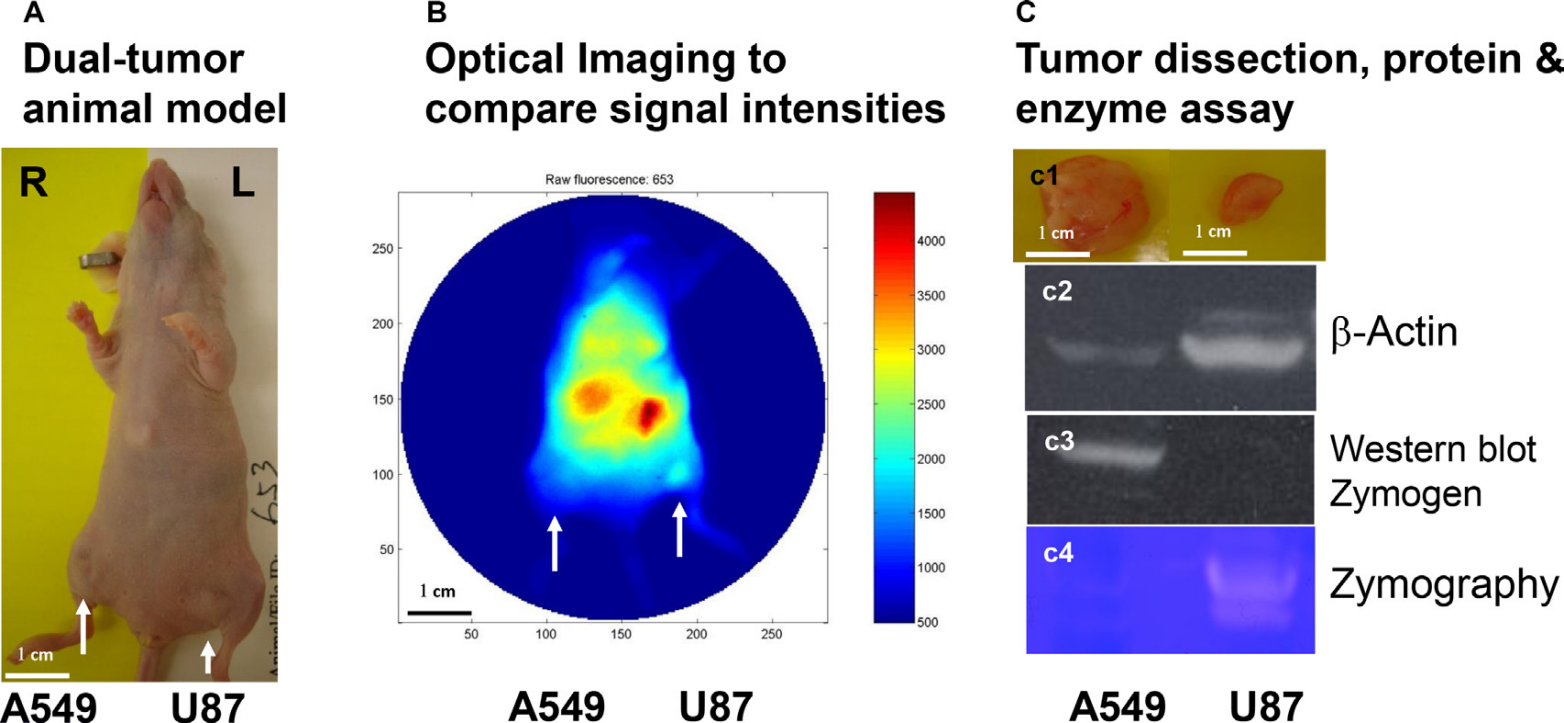
*In vivo* detection and western and zymographic analysis of zymogen and active MMP in two tumor types Female nude mice were inoculated with A549 and U87 cells, resulting in formation of two tumor masses (**A**, arrows). This dual-tumor model was used to minimize the effects of individual and temporal variability in subsequent analyses. The core structure conjugated to active–MMP-targeting peptide and IRDye800CW optical reporter was then intravenously injected. Image analysis showed that the U87 tumor had a higher level of active MMP than the A549 tumor, even though it was the smaller tumor (**B**). Comparison of the dissected tumors confirmed this size difference (**C1**). For western analysis and zymography, a 1.6 time amount of U87 than A549 protein from tumor tissue was loaded, as indicated by western analysis of β-actin (**C2**). Western analysis showed strong staining of zymogen in the A549 sample, but an undetectable level in the U87 sample (**C3**). Zymography showed a much higher level of active MMP in the U87 sample than A549 (**C4**).

We then applied the MMP-targeting agent in a dual-tumor mouse model of human glioma and lung carcinoma. A549 and U87 cells were inoculated into right and left legs of the mouse, respectively, of female nude mice, where tumor masses formed (Figure 6A). Optical imaging revealed a much higher level of signal intensity in the U87 than in the A549 tumor (Figure 6B). Comparison of the excised tumors showed that the U87 tumor was the smaller of the two, reflecting a slower growth rate (Figure 6C1). Although western analysis of β-actin in samples of U87 and A549 tumor protein loaded for western and zymographic analysis showed that a greater amount of U87 protein was loaded (Figure 6C2), the level of zymogen was much lower, and the level of active MMP was much higher in U87 than in A549 protein (Figure 6C3 and 6C4). The results of zymography show that the U87 tumor contains more active MMP than the A549 tumor.

**Figure 7:**
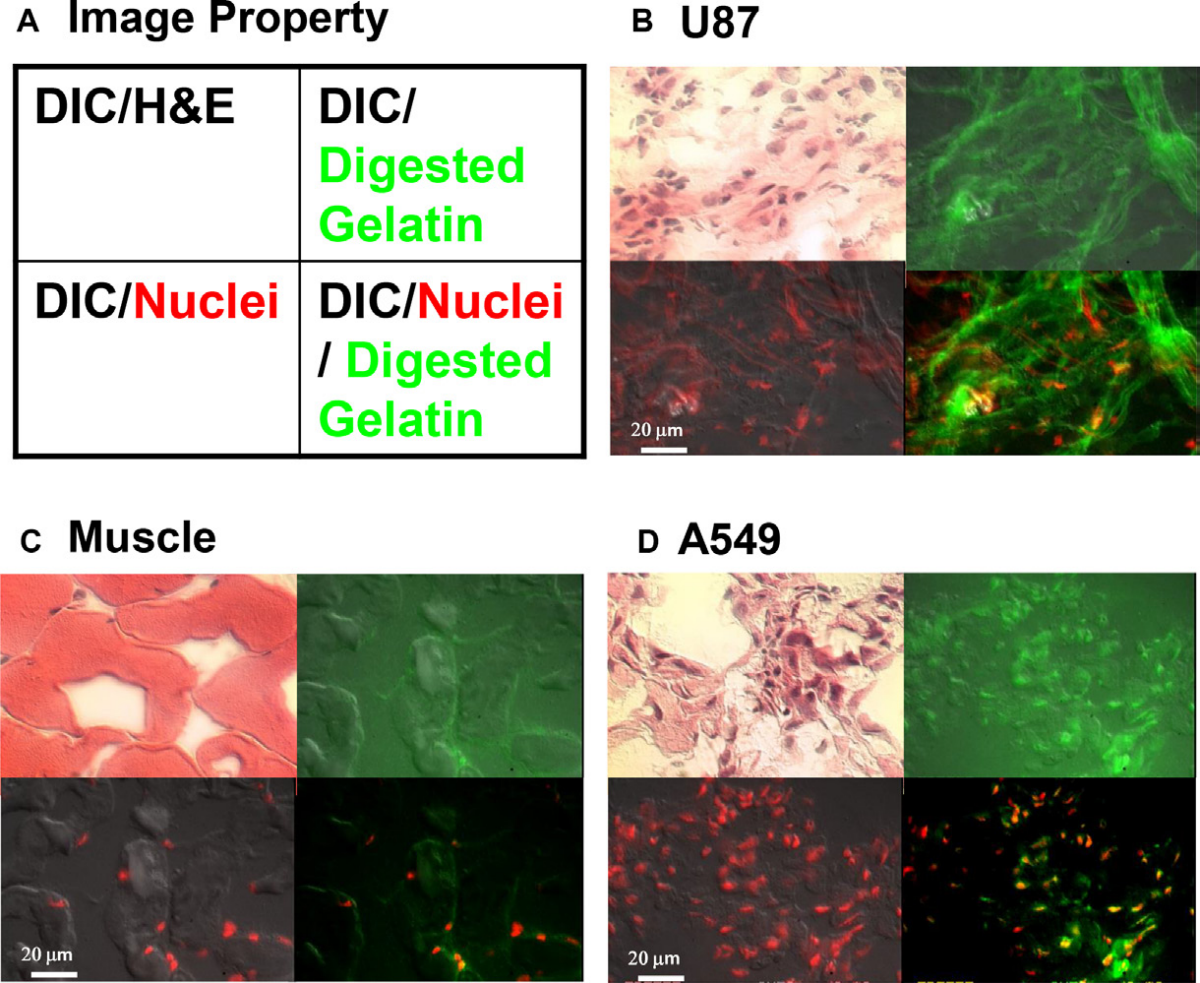
*In situ* zymography to detect active MMP at the cellular level Tissue sections were obtained from the tumors described in Supplementary Figure 3. (**A**) Graphic depicts processing of tissue shown in the sets of four images in panels (**B**–**D**). Each image was overlaid with different signal properties. Differential interference contrast (DIC) microscopy shows cell morphology, and hematoxylin and eosin (H & E) staining differentiates nucleus, cytoplasm, intracellular membranes, and extracellular matrix. Digested gelatin (green) represents MMP activity. The red signal represents the nucleus. (B) In sections of U87 tumor, H & E staining demonstrates the characteristic morphology of the glioma tumor. The diffuse green signal demonstrates strong MMP activity throughout the tissue section. The merged image showing nuclear staining and gelatin digestion demonstrates secretion and penetration of MMP into the tumor stroma. (C) The negative control muscle tissue section showed very weak MMP activity, which was entirely intracellular. (D) A549 lung cancer tissue showed less MMP activity, which was also entirely intracellular (yellow).

In order to determine the location of MMP in tumors, *ex vivo*, *in situ* tissue zymography was performed (Figure 7). Sections were analyzed by differential interference contrast (DIC) microscopy combined with hematoxylin and eosin staining, gelatin-digestion assay (*in situ* zymography) for detection of MMP activity, nuclear staining, or *in situ* zymography assay together with nuclear staining (Figure 7A). The results show the accurate co-localization of two components at the cellular level. Analysis of gelatin-digestion activity in U87 tumor sections showed that most enzymatic activity was in the tumor stroma (Figure 7B). Some muscle cells (negative control) also showed weak enzymatic activity (Figure 7C). A549 tumor tissue showed only very weak signals in cancer cells and stroma (green and yellow, Figure 7D) and no detectable signal in the stroma. Because the enzymatic activity was higher in the U87 tumor stroma than in the cells, we can conclude that this is indeed a secreted protein and our agent is specifically binding to the active enzyme, not the zymogen.

**Figure 8:**
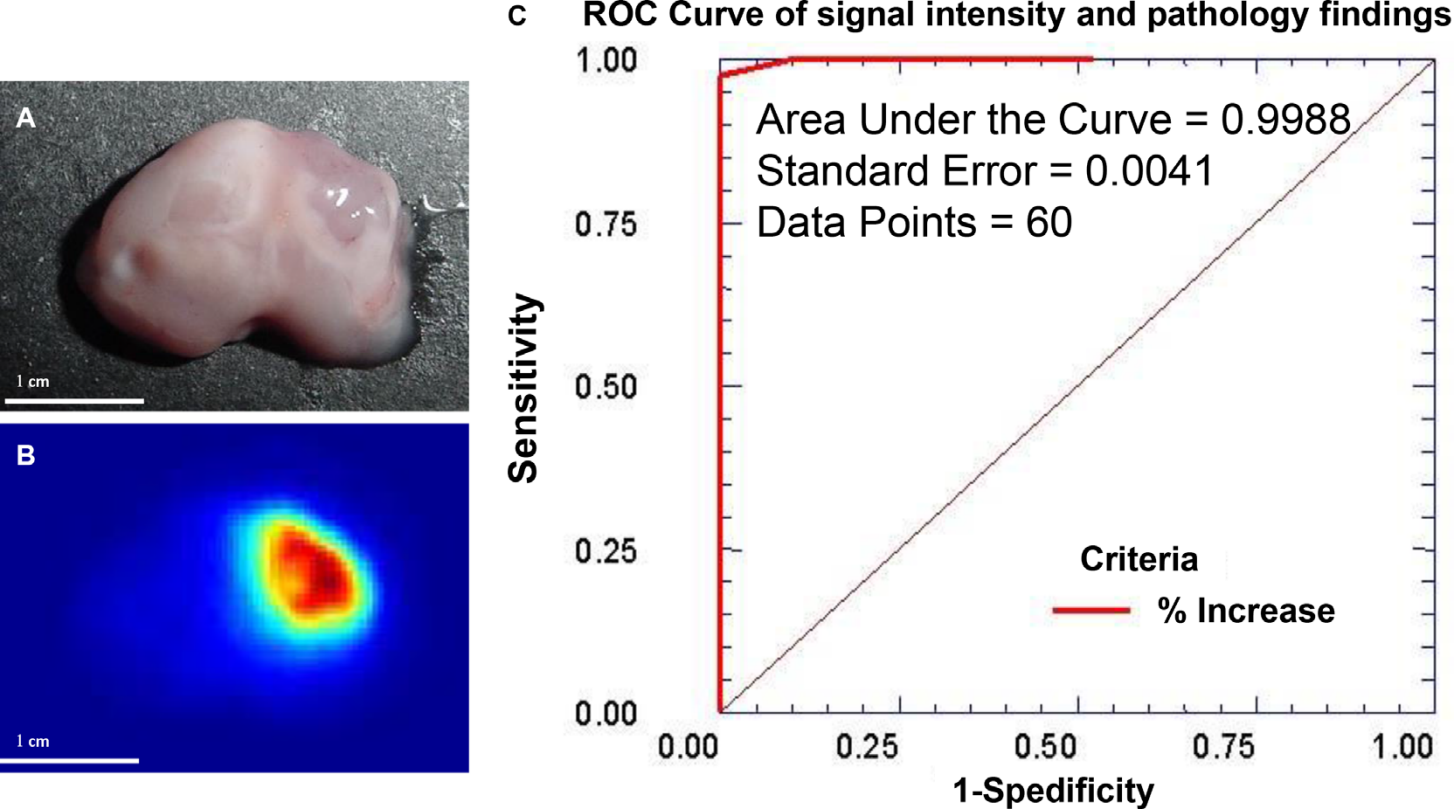
Change in MMP signal intensity can be used to determine sensitivity and specificity of the tumor cell detection Mouse was orthotopically inoculated with U87 human glioma cells and intravenously injected with MMP-targeting agent labeled with NIR reporter. The tumor was located by necropsy and excised (**A**). The intensity of the NIR reporter signal changed from high (red) to low (blue), and this change correlated with the tumor shape (**B**). Serial sections were prepared from tumor region based on signal intensity and sent for pathological analysis. An ROC curve was plotted using the results of pathological analysis as a gold standard and correlated to the increase in signal intensity in the image. With a total of 60 data points, the area under the curve (AUC) reached 0.9988 with a standard error of 0.0041 (**C**). Considering that the AUC is 1.00 for a perfect ROC curve, these data demonstrate that change in signal intensity provides excellent diagnostic guidance for real-time imaging-guided surgery.

Because a decreasing gradient of signal intensity was observed between the center of the tumor and peripheral normal tissue (Figure 5B), we used a mouse model of human glioma and generated a receiver operating characteristic (ROC) curve to test the sensitivity and specificity of tumor cell detection (Figure 8) and to determine U87 tumor margin. A mouse bearing a human U87 glioma was intravenously injected with MMP-targeting agent conjugated to NIR reporter. Tumor was removed (Figure 8A) and signal intensity recorded (Figure 8B). Using change in signal intensity as guidance, serial sections were prepared. The ROC curve was constructed using increase in signal intensity as continuous output and tumor cell appears as pathological finding. The ROC curve confirmed that this method yielded accurate, predictable results (Figure 8C).

**Figure 9:**
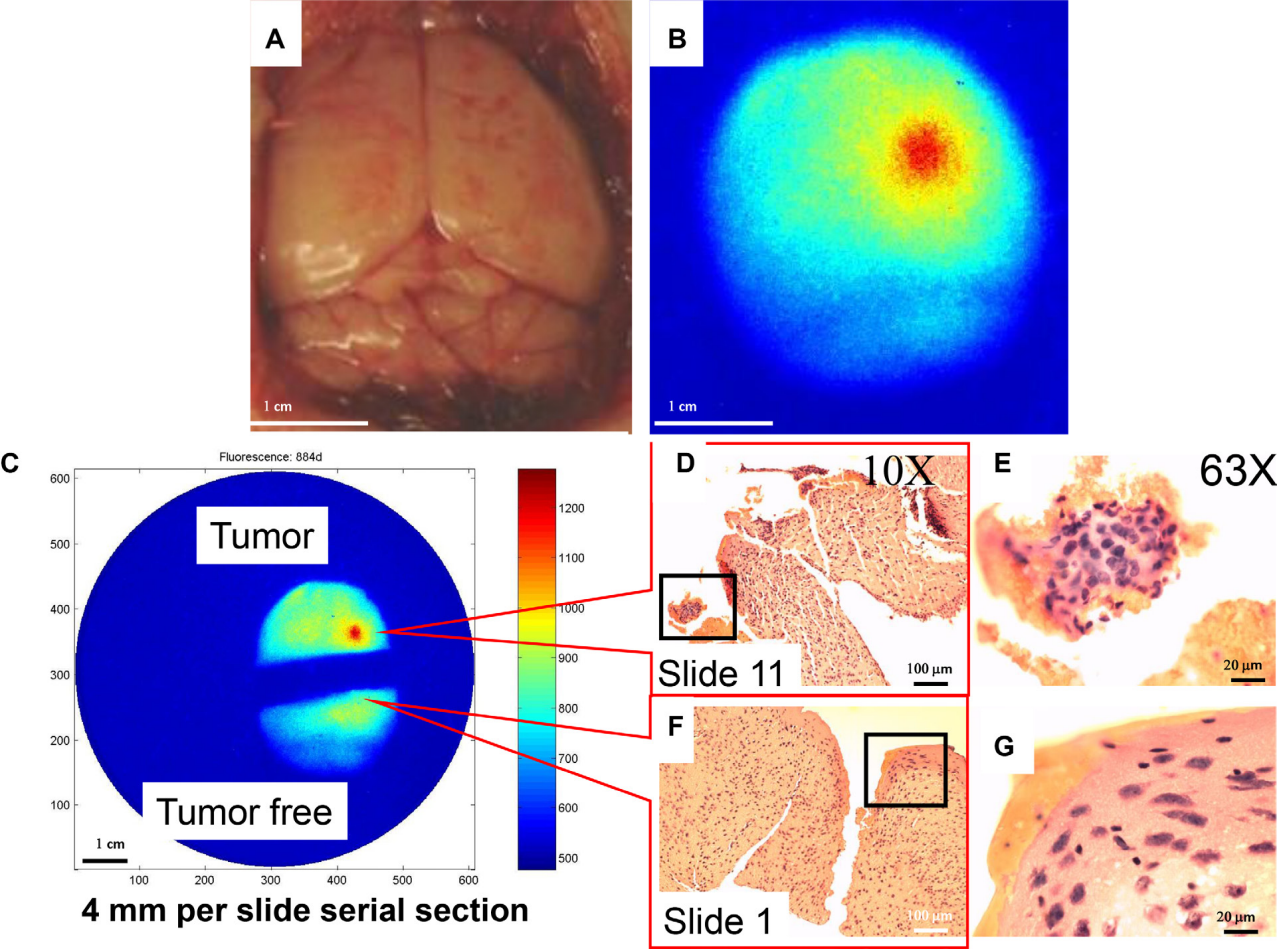
Double-blind, real-time imaging-guided surgery to generate tumor-free margin Ten animals were randomly assigned to receive either saline injection or U87 cell inoculation. All mice received the MMP agent through intravenous injection 3 days after the procedure. Brains were removed 24 h after injection of MMP agent. (**A**) The color image showed no obvious abnormality. (**B**) Near infrared (NIR) image clearly shows signal location. (**C**) Brain was bisected with a scalpel into regions of high and low signal intensity, and 4-μm serial sections were prepared from the indicated sites. The goal was to leave a tumor-free margin and retain as much normal brain tissue as possible, to preserve patient quality of life. (**D**) Tumor cells were observed on slide 11, 44 μm from the resection margin. This was confirmed by (**E**) examination of hematoxylin and eosin (H & E)-stained slides under a 63× objective. (**F**) No tumor cells were detected on slide 1 (F), as confirmed by examination of H & E-stained slides under a 63× objective (**G**).

Double-blind, real-time, imaging-guided surgery was performed 3 days after inoculation of U87 cells (Figure 9). Visual inspection of the intact brain revealed no abnormalities (Figure 9A). High signal intensity was detected in the right frontal lobe, with a gradient decrease in signal intensity (Figure 9B). The brain was bisected just outside the region of high signal intensity (Figure 9C), and slides containing single 4-μm serial sections were prepared. Tumor cells were first observed at slide 11, 44 μm from the dissection margin, in the tumor-bearing half of the brain (Figure 9D and 9E). Most importantly, no tumor cells were observed on slide 1 from the tumor-free half (Figure 9F and 9G). These data demonstrate that combined use of our agent and imaging-guided surgery may facilitate generation of a tumor-free resection margin and preservation of more normal brain tissue.

### Target-specific chemoradiotherapy and imaging

**Figure 10:**
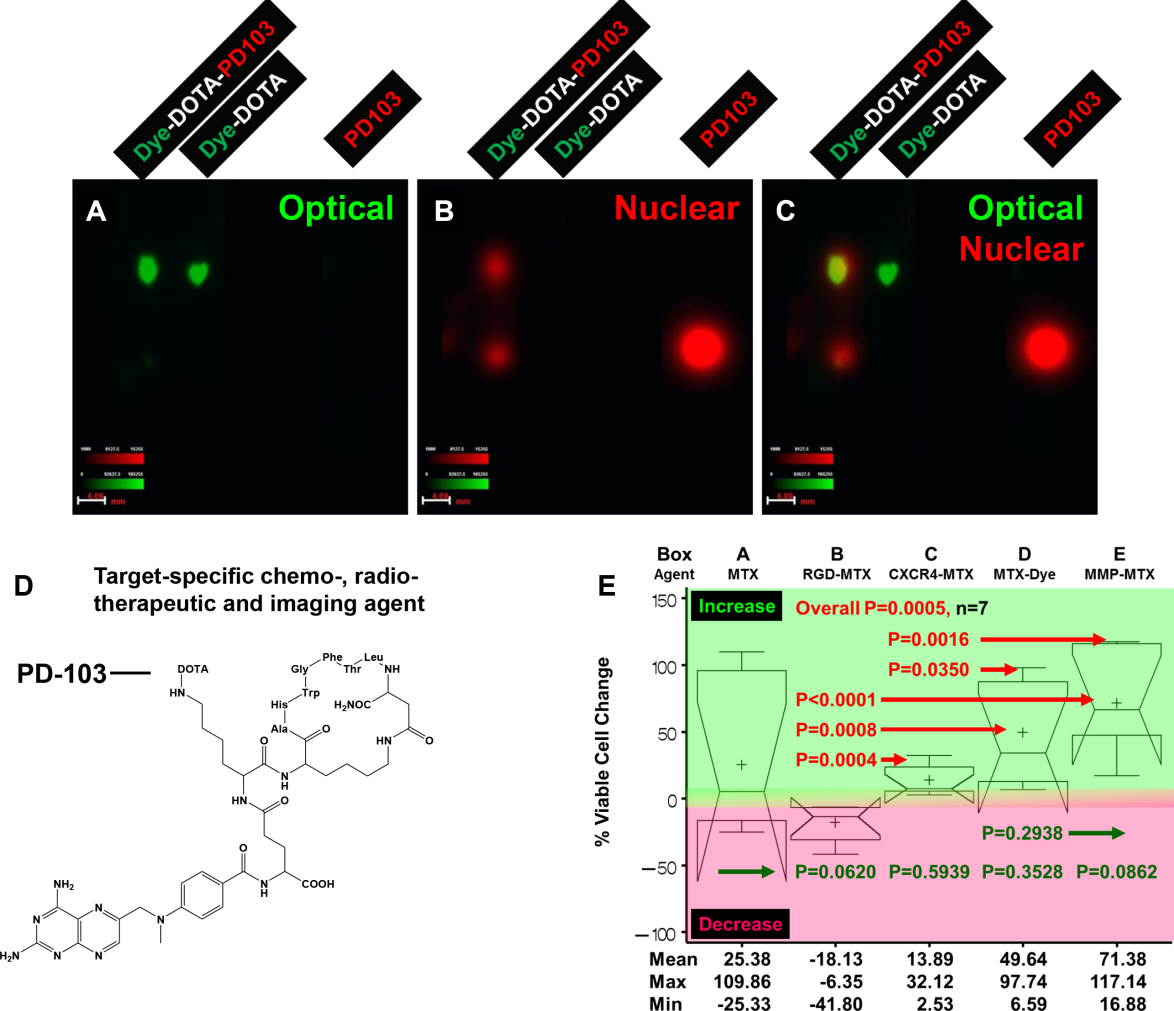
Three-armed core structure for target-specific chemoradiotherapy The core was conjugated to a targeting molecule, an optical reporter similar in structure to methotrexate (MTX) and isotope palladium (Pd-)103 through radiolinker DOTA. (**A**) The optical image shows that the distance migrated by the radiolabeled agent Dye-DOTA-PD103 and control compound Dye-DOTA was the same, demonstrating that the isotope did change the property of the complex. (**B**) The nuclear image shows that Dye-DOTA-PD103 migrated to a different distance than excess, unconjugated isotope and control free isotope. The presence of excess Pd-103 in the sample shows that the complex was completely labeled with isotope. The ratio of free isotope to the final triple labeled agent was close to 1:1. (**C**) The merge of images shown in panels (A) and (B) provided guidance for fine-tuning of the chemical reaction protocol. (**D**) Structure of targeted chemoradiotherapeutic and nuclear tracking agent is shown. (**E**) Percent change in viability of C2984 osteosarcoma cells after treatment with this agent conjugated to MTX alone (Box A) or to MTX conjugated to RGD (B), CXCR4 (C), dye (D), and MMP (E).

To test the feasibility of delivering chemotherapeutic and radiotherapeutic agents using a common targeting component, we designed and synthesized a dye with structure similar to that of the chemotherapeutic agent methotrexate (MTX). We further took advantage of isotope impurity and used the cytotoxic isotope palladium (Pd)-103 with both β- and γ -emitting as imaging agent. Figure 10 shows results of thin layer chromatography (TLC) analysis of the final product (Figure 10A–10C), the structure of the target-specific chemoradiotherapeutic and imaging agent (Figure 10D), and results of analysis of cytotoxicity of agent conjugated to MTX alone; to MTX conjugated to peptides targeting recognition sequence Arg-Gly-Asp (RGD), CXCR4, and MMP; and MTX conjugated to dye in human C2984 osteosarcoma cells (Figure 10E). To convert the agent to a target-specific chemoradiotherapeutic compound, the optical dye was replaced by MTX (Figure 10D). Increased cytotoxicity (decreased cell viability) results when cells are in the phase of the cell cycle in which the drug is effective, and vice versa. The RGD-targeted agent showed the greatest cytotoxicity (mean decrease in viability, 18.13%; range, 6.35%–41.80% (Figure 10E-Box B). The CXCR4-targeted agent showed significantly less cytotoxicity than the RGD-targeted agent (*P* < 0.0004), due in part to a lesser extent of internalization, but showed significantly greater cytotoxicity than MTX-dye or MMP-MTX agents (*P* = 0.035 and 0.0016, respectively) (Figure 10E-Box C). The complex lost its cytotoxic effects when the targeting component was replaced with dye (Figure 10E-Box D). (Figure 10E-Box D). The MMP-targeting compound had no effect (Figure 10E-Box E) because C2984 cells do not express MMP. All four MTX-conjugated agents showed less variability than MTX alone, suggesting that, unlike MTX, they are not antimetabolite drugs that rely on the cell cycle. These compounds will be cytotoxic only to cells expressing the targeted molecular markers, and not normal dividing cells, potentially showing less systematic toxicity to normal tissues and organs.

**Figure 11:**
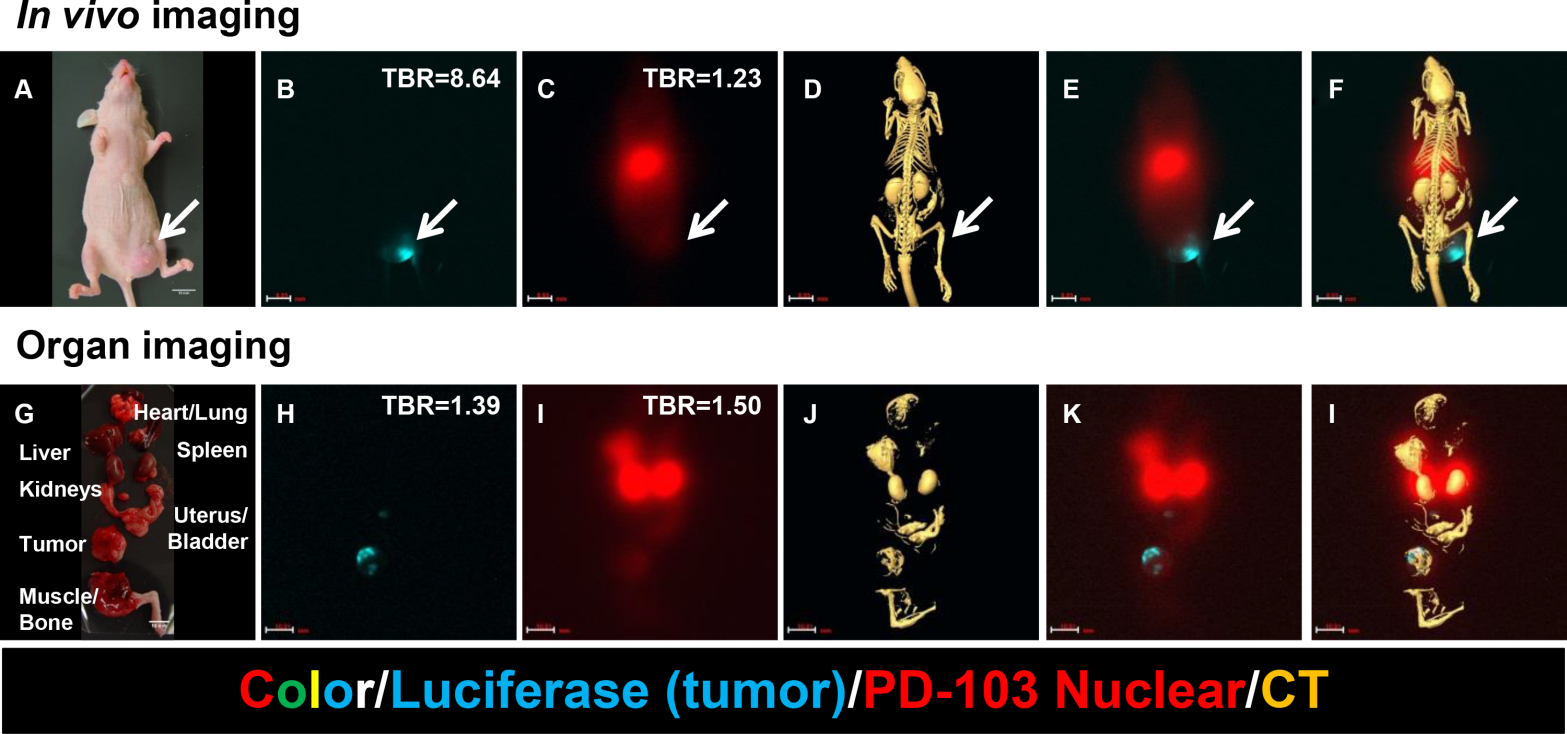
Chemoradiotherapeutic agent targeting MMP in a mouse model of breast cancer (**A**) Photograph shows tumor cell inoculation site (arrow) and size. (**B**) Precise tumor location is indicated by luciferase signal (blue). (**C**) Whole-body image shows agent distribution. (**D**) CT image shows anatomic structures. (**E**) Merge of images shown in panels (B) and (C) shows relative positions of tumor and agent. (**F**) Merge of images shown in panels (B), (C), and (D) shows tumor location and agent distribution relative to anatomic structures. It is clear that distribution of the therapeutic agent extends beyond the tumor itself to the region surrounding the tumor. Corresponding images of the dissected organs confirm the results of *in vivo* imaging (Figure 6g-l). (**G**) Organs were dissected to confirm *in vivo* imaging results. (**H**) Tumor location is indicated by luciferase signal (blue). (**I**) Palladium (Pd)-103 signal shows agent distribution in liver, kidney, bladder, and tumor. (J) CT image shows organ locations and X-ray densities. (**K**) Merge of images shown in panels (H) and (i) confirms presence of therapeutic agent in tumor region. (**L**) Merge of images shown in panels (H), (I), and (J) confirms distribution of agent in liver, kidney, bladder, and tumor.

To take advantage of isotope impurity and sensitivity of the imaging detector, we tracked the whole-body distribution of the agent in a tumor-bearing animal model. *In vivo* images show tumor location (Figure 11A and 11B), whole-body distribution of the agent (Figure 11C), anatomic structures (Figure 11D), and overlay of these parameters (Figure 11E and 11F). Corresponding images of the dissected organs confirm the results of *in vivo* imaging (Figure 11G–11I).

## DISCUSSION

Precision medicine is the trend in research and clinical practice and entails accurate disease prediction, diagnosis, effective treatment with minimal side effects, and therapeutic management [6]. Disease prediction is challenging because, of all the factors and potential interactions involved in initiation of disease, only the genetic component is a known entity. Genetic mutation is a normal component of evolution and disease, thus, accurate prediction of a disease based purely on a patient's genetic profile is a great challenge in modern medicine. Unlike disease prediction, progress in molecular medicine in the last 15 years has made possible accurate diagnosis, treatment, and disease management. Target-specific agents allow detection of specific disease markers, enabling accurate diagnosis and targeted delivery of therapeutic agents.

There are many ways to combine target, diagnostic, and therapeutic modalities in a single agent. We designed our agents based on the three-armed core structure because 1) it constitutes a universal structure for use in diagnosis, therapy, and long-term management amenable to the needs of different patient populations; 2) the manufacturing process is simple and economical for a low-budget laboratory or large-scale application; 3) the final product is consistent in quality and of high purity; 4) it is a small molecule and has little impact on the performance of the final agent in terms of pharmacodynamics, pharmacokinetics, or any of the agent's intended functions; 5) it is sufficiently stable for long-term storage, increasing availability and reducing variability in agent quality; 6) the design is flexible and adaptable to different target, diagnostic, and therapeutic modalities; and 7) it displays a one-to-one ratio of targeting to therapeutic/detection components to facilitate therapeutic dosing and quantitative diagnosis.

The disadvantage is delivering fewer payloads to the diseased cells. However, we believe a target-specific approach is more important in order to minimize the systematic toxicity [7–8] and the therapeutic efficacy can be increased by deliver different drug with strong cytotoxic effects. Since most diseases have multiple markers and one target-specific agent can only solve one problem, accurate evaluation will help guide precise treatment plan. Moreover, as disease markers change during disease progression, the duty of precision medicine is to detect these changes early and apply a new agent to individual patient, as well as at one specific time-frame, as needed. The therapeutic strategy will change from the current “treat disease with what we have' to the future precision medicine that “treat disease according to what they have (molecular markers or genetic characteristics) and/or what they need (nutrients, metabolic compounds, or growth factors)”.

**Figure 12:**
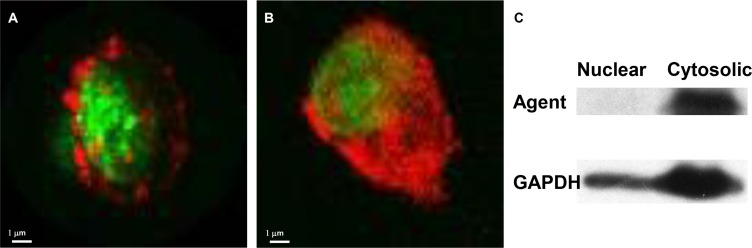
Cellular location of agent changes with change in targeting component on the core structure Confocal microscopic images show the optical labeled Herceptin agent located at the cell membrane (**A**) and in the cytosol after changing the target component to a peptide (**B**). Western analysis confirmed the location of the agent in the cytosolic, but not the nuclear, compartment (**C**).

**Figure 13:**
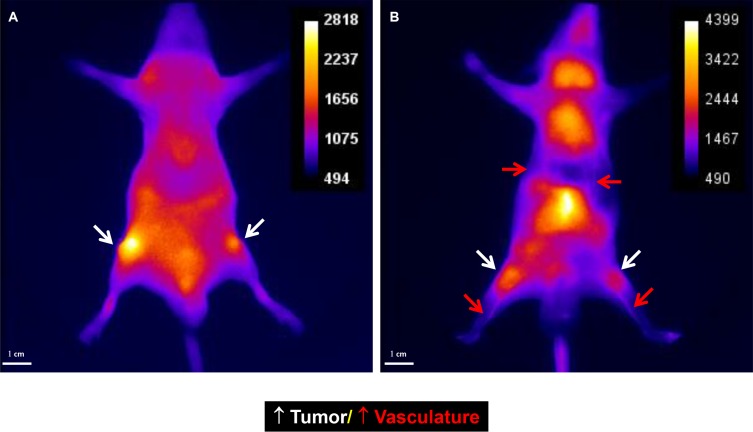
Peptides the labeled with optical dye are flexible targeting components Alteration of one amino acid can change the destination of the agent. The original peptide sequence targeted the tumor mass (**A**). After alteration of one amino acid, the agent targeted the vasculature (**B**). Further, the vasculature-targeted agent is capable of gelling and forming an embolus. Gel formation can be easily controlled by the modulating injection speed. Slow injection is used for vasculature imaging and rapid injection for vasculature block. Such an agent may be useful in treating tumors or aneurysms.

We have used this core structure to generate multiple target-specific agents, including peptides [4, 9–13], antibodies [14–15], cytokines and chemokines [4, 12–13] and small-molecule compounds [7, 16–7]. We consider peptides ideal targeting components. They are easy to synthesize and conjugate directly to the core. A small peptide designed to bind to the most highly conserved region of a target will retain its binding capacity even when the target begins to mutate. Changing the sequence of a peptide readily alters its targeting destination [18–19] and determines whether the agent remains at the cell surface or is endocytosed (Figure 12). Figure 13 demonstrates this flexibility of peptide targeting components and shows that alteration of one amino acid can change the destination of the agent from tumor to vasculature. Replacement of a single amino acid in a targeting moiety allows one chemist to generate hundreds agents with unique binding properties annually. Another advantage of peptide agents is they will not stimulate or activate the disease markers, and any agent that has potential to stimulate or activate occult disease should be avoided [20].

For radiotherapy, the nuclear arm can be conjugated to different nuclear chelating agents in order to conjugate different isotopes. Based on our previously described imaging agents [4, 10, 14, 21–23], it is possible to generate other radiotherapeutic agents using impurity isotopes and provide a good tracking reporter for PET or SPECT. Isotopes that do not target specific organs should be selected, avoiding isotopes such as iodine and technetium. Free excess isotope will be rapidly eliminated from the body through the urinary tract with minimal impact on diagnosis or toxicity.

**Figure 14:**
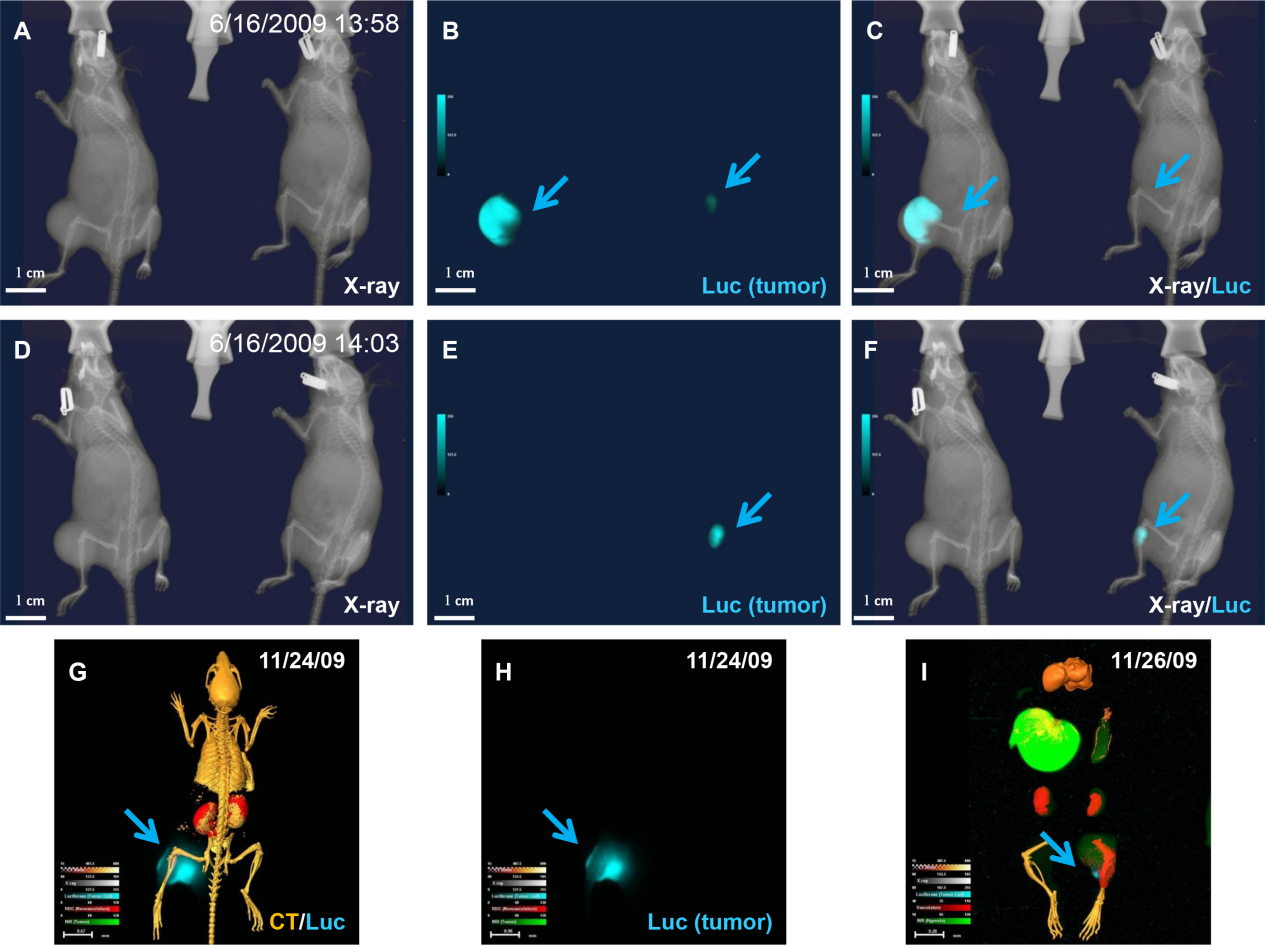
Variability of enzymatic reaction (**A**–**F**) X-ray (A and D), luciferase (B and E), and merged (C and F) images of mice bearing luciferase-positive tumor cells captured at an early time point (A). (B) Luciferase imaging shows different signal intensity (arrows) in the two animals. Both tumors showed strong signal under ideal enzyme reaction conditions. (C) The merged image shows whole body structure and tumor location. (D) Animal on left inhaled CO^2^ for 5 min, followed by additional X-ray imaging with animal on right serving as control, and (E) luciferase signal disappeared in CO^2^-treated animal. (F) Merged image confirmed the total disappearance of signal under hypoxic conditions. (**G**) Animal was sacrificed by Isoflurane overdose under continuous oxygen supply and dissected organs and tissues were preserved at −80°C. (**H**) Image clearly shows luciferase signal remains after 2 days under stable storage conditions. The uneven luciferase signal in the tumor demonstrated nonuniform luciferase expression and/or enzymatic reaction. (**I**) Enzymatic activity was preserved at −80°C for 2 days.

The most common therapeutic approaches to cancer are surgery, chemotherapy, and radiation. Of these, surgery presents the greatest technical challenge. However, despite recent advances in surgical instrumentation, lack of a molecular beacon at the tumor margin constitutes a bottleneck. In general, combination therapy allows better patient management than monotherapy, and the goal of this study was to use a single targeting component with flexible combinations of therapeutic components. However, the approach described here is not without pitfalls. Figure 14 provides an example of how a single factor, hypoxia, can inhibit the luciferase-luciferin reaction and confound interpretation of assay results. Enzymatic reactions require optimization of multiple factors, any of which might change with disease condition/progression, and reliance on these reactions should be undertaken with caution.

In addition to the capacity of surgery to eliminate disease, chemo- and radiotherapy rely on a balance between local concentration of therapeutic agent and systematic toxicity. However, no disease marker is uniformly expressed during disease development, progression, and treatment, and biological features of solid tumors can prevent penetration of therapeutic agents [16]. Therefore, development of multiple target-specific agents for precision medicine is warranted. Our data demonstrate the feasibility of using a small-molecule core structure for this purpose. We also think administration of multiple target-specific agents will be a better way to increase local therapeutic dose. The disadvantage of this approach is that continuous evaluation of biomarker status and adjustment of therapeutic reagents is required. A limitation of this study is that it does not address patient survival and quality of life, which should be addressed in future studies. Our human clinical trial results demonstrate that an approach that induces a majority of disease cells to enter a particular phase of the cell cycle or express a specific biomarker, followed by application of our class of target-specific agent, might improve both survival time and quality of life [24].

In conclusion, we present a simple small-molecule core structure with three arms for diagnostic, detection, and therapeutic purpose. The target arm can be conjugated to an antibody, peptide, cytokine, chemokine, or small-molecule compounds. The other two arms can be modified with different moieties, so that the function of the final agent can range from pure target-specific optical/nuclear diagnostic imaging agent to a pure target-specific chemoradiotherapeutic agent. By switching the combination of these two arms, the core structure can become an agent with “seek, treat, and see” capabilities. The flexible combinations can be adapted to different patient populations (adult or pediatric) and disease treatments (surgery, chemotherapy, radiotherapy, or a combination of any two of these). By maintaining the same target component, this core structure will link diagnostic and therapeutic components for application in precision medicine.

## MATERIALS AND METHODS

### Chemicals and synthesis

All compounds were designed and synthesized in-house, as previously described in detail [4, 7, 10–12, 14, 15, 17, 21–23, 25], purified by high-performance liquid chromatography (HPLC) and confirmed by mass spectrometry and analytical HPLC. The nuclear agents were tagged with palladium-103 (Nordion, Vancouver, Canada) or yttrium-90 (PerkinElmer Life and Analytical Sciences, Billerica, MA). The most difficult step in the chemical synthesis is the radiolabeling procedure. Variation in isotope impurity greatly influences labeling efficiency, making it difficult to achieve a perfect one-to-one ratio between the nuclear and targeting/chemotherapeutic arms. Further, we always select isotopes that do not target specific organs, avoiding isotopes such as iodine and technetium, which concentrate in thyroid and bone, respectively.

### Cell lines

Human U87 glioma, A549 lung carcinoma, and F4 and C2984 osteosarcoma cell lines were purchased from the American Type Culture Collection (Manassas, VA) and cultured in Dulbecco's Modified Eagle Medium with high glucose or F12 medium (Invitrogen, Carlsbad, CA) supplemented with 10% fetal bovine serum (Hyclone, Logan, UT) at 37°C in a humidified atmosphere of 5% CO_2_.

### Tumor xenografts

Female nude mice (4– to 6-weeks old, 18–22 g) (Harlan, Indianapolis, IN) were maintained in pathogen-free mouse colonies in facilities accredited by the American Association for Laboratory Animal Care, and all experiments were performed in compliance with the guidelines of the Institutional Animal Care and Use Committee. For tumor implantation, tumor cell cultures were harvested near confluence by treating monolayers with 0.05% trypsin-EDTA. Cells were centrifuged at 130 × g for 5 min and resuspended in sterile phosphate-buffered saline. Approximately 1 × 10^6^ cells were implanted into each mouse.

### Confocal microscopic imaging

Stained cells were transferred to slides for microscopic examination. Images were captured with an Olympus Fluorview 1000 confocal microscope (Olympus America, Center Valley, PA). Signal intensities were recorded from one slice of multiple z-stacks with 0.5-μm gaps.

### Animal imaging

Tumors developed to 8–15 mm in diameter after 3–4 weeks of inoculation in the mice. Agents (2–10 nanomols) were injected into the tail veins of anesthetized mice. Mice were imaged immediately after injection and for as long as 48 h afterward. Optical and x-ray images were recorded by Kodak *In-Vivo* Multispectral System FX (Carestream Health Molecular Imaging, New Haven, CT). *In vivo* positron emission tomography (PET)/single photon emission computed tomography (SPECT)/computed tomography (CT) imaging was performed on a Siemens MicroCAT II SPECT/CT and Inveon PET instruments (Siemens Medical Solutions, Malvern, PA).

### Statistical analysis

SAS software v9.4 (SAS Institute, Cary, NC) was used to analyze data by one-way ANOVA or the general linear model. Data comparison was presented in notched box-and-whisker plots. The medians (central lines) of two box-and-whisker plots were considered to be significantly different at the 0.05 level (95% confidence) if the corresponding notches did not overlap.
